# Polygenic risk scores and Parkinson’s disease in South Africa advancing ancestry informed disease prediction

**DOI:** 10.1371/journal.pgen.1012064

**Published:** 2026-03-09

**Authors:** Kathryn Step, Carene Anne Alene Ndong Sima, Spencer Grant, Jonggeol Jeffrey Kim, Emily Waldo, Soraya Bardien, Ignacio F. Mata, on behalf of the Global Parkinson’s Genetics Program (GP2)

**Affiliations:** 1 Division of Molecular Biology and Human Genetics, Faculty of Medicine and Health Sciences, Stellenbosch University, Cape Town, South Africa; 2 South African Medical Research Council Centre for Tuberculosis Research, Stellenbosch University, Cape Town, South Africa; 3 Center for Alzheimer’s and Related Dementias, National Institute on Aging and National Institute of Neurological Disorders and Stroke, National Institutes of Health, Bethesda, Maryland, United States of America; 4 Department of Clinical Neurosciences, School of Clinical Medicine, The University of Cambridge, Cambridge, United Kingdom; 5 Department of Molecular and Human Genetics, Baylor College of Medicine, Houston, Texas, United States of America; 6 Genomic Medicine, Lerner Research Institute, Cleveland Clinic Foundation, Cleveland, Ohio, United States of America; 7 South African Medical Research Council/Stellenbosch University Genomics of Brain Disorders Research Unit, Stellenbosch University, Cape Town, South Africa; Newcastle University, UNITED KINGDOM OF GREAT BRITAIN AND NORTHERN IRELAND

## Abstract

Parkinson’s disease (PD) is a complex neurodegenerative disorder with environmental and genetic influences. Using genotyping array data of 661 South African PD cases and 737 controls, we conduct a polygenic risk score (PRS) analysis using PRSice-2. Summary statistics from two PD association studies have been used as base datasets. We split the target dataset into training (70%; n = 979) and validation (30%; n = 419) cohorts. We test various clumping window sizes, linkage disequilibrium thresholds, and *p*-value thresholds to determine the optimal combination for risk prediction. Additionally, we investigate the variance explained by different combinations of covariates. Overall, we observe modest predictive performance (AUC: 0.5847-0.6183). Age at recruitment emerges as the strongest individual predictor, while sex contributes the least. These findings provide the first evaluation of PRS performance for PD in a highly admixed South African cohort, underscoring the importance of including underrepresented populations in genetic risk prediction. By systematically assessing predictive performance across two base datasets, we highlight how ancestry composition and study design affect risk estimation in diverse populations. This work lays a foundation for refining genomic prediction in admixed populations and contributes to ongoing efforts to ensure that advances in precision medicine are globally relevant.

## Author summary

Parkinson’s disease is a complex brain disorder influenced by both genes and environment. Most of what we know about the genetic contribution to this disease comes from studies in people of European ancestry, leaving major gaps in our understanding of how genetic risk works in other populations. In this study, we examined how well existing genetic risk models, known as polygenic risk scores, can predict Parkinson’s disease in people from South Africa, a population with a rich mix of ancestries. We compared different approaches to building these scores and tested how accurately they could identify individuals with the disease. Although we found that genetic scores alone are modest predictors compared to age, our results highlight important insights about the limits and possibilities of using genetic information in diverse populations. By including underrepresented groups in genetic research, our study takes an important step toward making precision medicine more inclusive and equitable worldwide.

## Introduction

Parkinson’s disease (PD) is a complex neurodegenerative disorder characterized by motor and non-motor symptoms [1]. Once considered primarily sporadic and environmentally driven [2,3], research has revealed a multifactorial etiology involving monogenic causes, gene-environment, and gene-gene interactions [2]. Approximately 5–10% of cases are monogenic, caused by single-gene mutations with large effect sizes, identified through genetic linkage analysis in families [4,5]. Although often observed in familial PD, they can occur in sporadic cases due to incomplete penetrance [6]. Genome-wide association studies (GWAS) have further shown that sporadic PD has a polygenic basis, with many small-effect variants jointly contributing to risk [7].

These susceptibility variants can be used for risk prediction through polygenic risk scores (PRS) [8]. In a PRS analysis, the variants identified through GWAS, along with their effect sizes (whether conferring increased or decreased risk) are combined to estimate an individual’s genetic predisposition to a disease [9]. In 2016, the first report on polygenic risk and clinical outcomes for PD was published [10]. Since then, several studies have evaluated PRS for PD risk prediction, with predictive performance, assessed by the area under the receiver operating characteristic curve (AUC), ranging from ~60%-76% [7,11–14]. This is largely dependent on the number of single-nucleotide polymorphisms (SNPs) included and the population characteristics [15].

PRS analysis classifies individuals by relative disease risk, supporting risk stratification, early intervention strategies, and tailored precision medicine approaches [8,15]. We conducted the first PRS analysis for PD in a South African cohort.

## Results

### PRSice-2 for disease status prediction

The analysis included 35,075,375 variants in the two target datasets: training (70%; n = 979) and validation (30%; n = 419) cohorts. We conducted the analysis using two sets of summary statistics as the base data. For each, we evaluated varying combinations of thresholds for SNP inclusion (**S1** and **S2 Figs**). The analyses were performed on the training dataset to determine the optimal thresholds and replicated in the validation dataset. All analyses were adjusted for sex, age, and inferred local ancestry components (ANC) with linkage disequilibrium (LD) estimated using the European (EUR) reference panel from the 1000 Genomes project.

One of the main uses of PRS is to predict case status according to their genetic risk or predisposition, making it a useful prognostic tool (**S3 Fig**) [15]. Using the EUR-based summary statistics [7], we tested 48 combinations of parameters to identify the best-performing PRS model for PD status prediction (**S4 Fig**). The highest predictive performance was observed under the following parameter set: 100kb clumping window, *r²* = 0.8, and a SNP inclusion threshold of *p*-value = 1 × 10^−3^. Here, 3,466 SNPs remained after clumping and the full R^2^ explained 35.84% of the variance in the disease phenotype (PRS R^2^ = 0.005), with a strong association (empirical *p*-value = 0.076). The PRS analysis was conducted on the validation dataset (30%) using the abovementioned parameter combinations and the 3,466 SNPs included in the model. When applied to the validation set, 37.90% of the variance in case-control status (PRS R² = 0.019).

Given the high genetic admixture in the South African cohort [16], we evaluated predictive accuracy using multi-ancestry summary statistics from Kim *et al*. (2024) [17]. We tested the same 48 parameter combinations as for the previous analysis (**S5 Fig**). The highest predictive performance was observed under the following parameter set: a 100kb clumping window, *r²* = 0.8, and a *p*-value threshold of 1 × 10^−3^. The full R^2^ explained 36.93% of the variance (PRS R² = 0.015; adjusted R² = 0.016; *p*-value = 9.82 × 10^−5^; empirical *p*-value = 5.0 × 10^−4^). A total of 3,208 variants remained after clumping in this model. We performed PRS analysis on the validation dataset (30%), where the best-fitting model, defined by a *p*-value threshold of 1 × 10^−3^ and including 3,208 SNPs, explained 40.17% of the variance in disease phenotype (PRS R² = 0.042).

### Assessment of model performance

**Fig 1:**
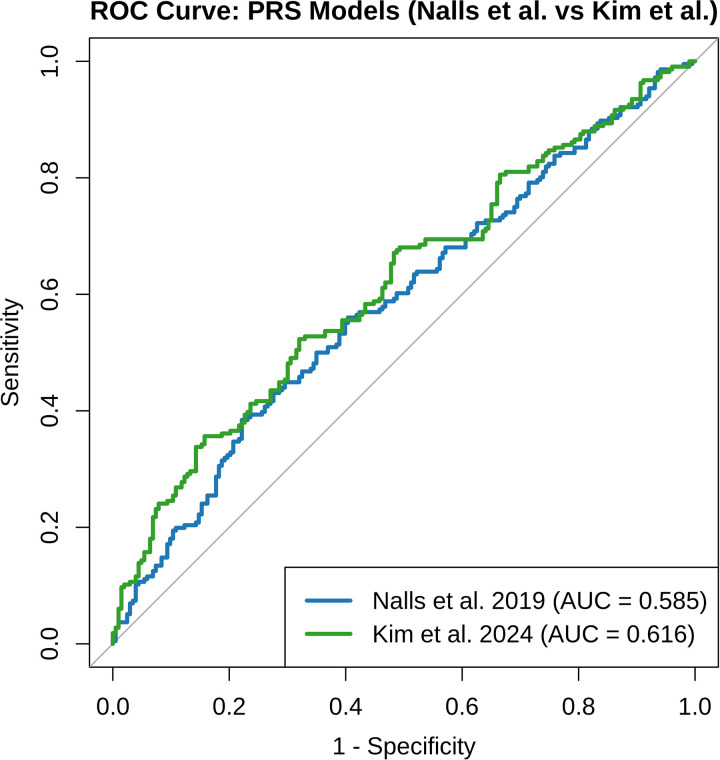
Receiver Operating Characteristic (ROC) Curve Comparing Polygenic Risk Score (PRS) Models for Case Status. The ROC curves compare the predictive performance of two PRS models for Parkinson’s disease: one based on Nalls *et al*. (2019) (blue) and the other on Kim *et al*. (2024) (green). The area under the curve (AUC) indicates the discriminative ability of each model, with higher AUC values reflecting better classification of cases and controls. The AUC for the Nalls model is 0.585, while the AUC for the Kim model is 0.616. PRS were calculated and matched to phenotype data from the same sample set (N = 419).

The AUC, sensitivity, and specificity were assessed using the two base datasets as well as the training and validation cohorts of the target dataset (**Table 1**; Fig 1). We assessed the mean AUC (Nall *et al* 2019: 0.6038 ± 0.0103; Kim *et al* 2024: 0.6052 ± 0.0178) across 20 random data splits to assess the robustness of our data split into training and validation cohorts. The results were consistent with the original split presented below, indicating the predictive performance was stable and not notably influenced by the random split.

**Table 1:** Model performance across base and target datasets.

Base and target data	Nalls with training	Nalls with validation	Kim with training	Kim with validation
**Best p-value threshold**	1.00E-05	5.00E-85	3.60E-04	5.00E-04
**PRS.R2**	0.0047	0.0194	0.0151	0.0421
**Full.R2**	0.3584	0.3790	0.3693	0.4017
**Null.R2**	0.3535	0.3540	0.3535	0.3600
**Coefficient**	0.0207	0.1129	0.0621	0.0947
**Standard Error**	0.0099	0.0396	0.0159	0.0228
**PRS association p-value**	0.0359	0.0043	9.82E-05	3.24E-05
**AUC (95% CI)**	0.6077 (0.5721-0.6433)	0.5847 (0.5302-0.6391)	0.6183 (0.5830-0.6537)	0.6159 (0.5624-0.6694)
**Accuracy (95% CI)**	0.5700 (0.5383-0.6012)	0.5585 (0.5095-0.6067)	0.6016 (0.5702-0.6325)	0.5823 (0.5335-0.6300)
**Balanced Accuracy**	0.5343	0.5560	0.5856	0.5805
**Sensitivity**	0.1416	0.6343	0.4090	0.6388
**Specificity**	0.9270	0.4778	0.7622	0.5222

Legend: The DeLong p-value for the training dataset is 0.9778 and for the validation dataset is 0.7057. Accuracy, balanced accuracy, sensitivity, and specificity were calculated using a 0.5 threshold. AUC values correspond to the PRS-only model derived from ROC curves; details on the PRS + covariates model are provided in the Methods. PRS R² reflects the variance in disease liability explained by the PRS (liability scale, assuming 10% prevalence), and PRS R² applies Nagelkerke’s adjustment for ascertainment. Full R² and Null R² represent variance explained by the full model (PRS + covariates) and covariates only, respectively. The “Coefficient” and “Standard Error” refer to the PRS term in the logistic regression model. The SNP inclusion p-value threshold was determined in the training dataset, and the resulting SNP set was applied unchanged in the validation dataset. The p-values reported reflect the association between the polygenic score and disease status within each dataset. Empirical p-values are based on 10,000 permutations. The number of SNPs refers to variants retained after clumping. Full explanations of all metrics are provided in the Methods section.

Using the Nalls *et al*., 2019 summary statistics, the PRS demonstrated a moderate ability to distinguish between PD status, with an AUC of 0.6077 (95% CI: 0.5721-0.6433) in the training dataset and 0.5847 (95% CI: 0.5302-0.6391) in the validation dataset. At a fixed probability threshold of 0.5, classification accuracy was 57.00% (95% CI: 0.5383-0.6012) in the training dataset and 55.85% (95% CI: 0.5095-0.6067) in the validation dataset. The observed balanced accuracy varied slightly between datasets (53.43% and 55.60%). Sensitivity was higher in the validation dataset (63.43%) compared to the training dataset (14.16%), whereas specificity was higher in the training dataset (92.70% versus 47.78%).

In contrast, the PRS model constructed using summary statistics from Kim *et al*., 2024 yielded a comparable discriminative ability, with an AUC of 0.6183 (95% CI: 0.5830-0.6537) in the training dataset and 0.6159 (95% CI: 0.5624-0.6694) in the validation dataset. Classification accuracy at the 0.5 threshold was 60.16% (95% CI: 0.5702-0.6325) and 58.23% (95% CI: 0.5335-0.6300) in the training and validation datasets, respectively. Balanced accuracy was modest in both datasets (58.56% and 58.05%), with sensitivity again higher in the validation dataset (63.88%) compared to the training dataset (40.90%). Specificity followed the same pattern as observed in the EUR-based base dataset [7], with higher values in the training dataset (76.22%) relative to validation (52.22%).

Finally, we evaluated the predictive performance of the PRS using top percentile thresholds (S1 **Table**). The sensitivity increased as more cases were included when the threshold was lowered, while specificity decreased correspondingly. The positive predictive value remained low across all thresholds, whereas the negative predictive values were consistently high (>99%). The overall patterns observed were similar between the PRS derived from the two base datasets with minor differences in the number of cases captured at the top 5% threshold.

### Covariate contribution to the variance observed

We evaluated the contribution of covariates to the explained variance by examining their effect on model performance (**Table 2**). We looked at the two variance models (Full R^2^ and Null R^2^) under seven different covariate inclusion scenarios, using each possible combination of covariates: age, sex, and ANC.

**Table 2:** Variance explained by polygenic risk score models with different covariate adjustments for the training dataset.

PRS R2	PRS R2 adjusted	Full R2	Null R2	Empirical p-value	Covariates included
**Base dataset: Nalls *et al*., 2019**					
0.0047	0.0049	0.3584	0.3535	0.0761	AGE, SEX, ANC
0.0297	0.0296	0.3107	0.2811	9.99E-05	AGE
0.0317	0.0256	0.0324	0.0068	9.99E-05	SEX
0.0116	0.0096	0.0660	0.0563	0.0158	ANC
0.0275	0.0276	0.3199	0.2923	9.99E-05	AGE, SEX
0.0055	0.0057	0.3471	0.3414	0.5159	AGE, ANC
0.0112	0.094	0.0732	0.0638	0.0178	SEX, ANC
**Base dataset: Kim *et al*., 2024**					
0.0151	0.0158	0.3693	0.3535	5.00E-04	AGE, SEX, ANC
0.0215	0.0213	0.3024	0.2811	9.99E-05	AGE
0.0552	0.0448	0.0517	0.0068	9.99E-05	SEX
0.0415	0.0349	0.0913	0.0564	9.99E-05	ANC
0.0224	0.0224	0.3147	0.2923	9.99E-05	AGE, SEX
0.0139	0.0144	0.3558	0.3414	0.1100	AGE, ANC
0.0433	0.0367	0.1004	0.0638	9.99E-05	SEX, ANC

Legend: Table shows PRS R² values, adjusted and unadjusted, with corresponding full and null model R², empirical significance, and number of SNPs included, stratified by covariates used in each model. PRS R2 is the variance explained by the PRS. PRS R2 adjusted is the PRS R2 adjusted for ascertainment. The Full R2 is the variance explained by the full model (including the covariates). Null R2 is the variance explained by the covariates only. PRS R2 and adjusted R2 values are rounded to four decimal places; values that appear identical may differ at higher precision and any apparent discrepancies are due to rounding. ANC, ancestry; PRS, polygenic risk score; SNPs, single nucleotide polymorphisms.

We assessed this using the Nalls *et al*., 2019 summary statistics as the base data. Here, the highest PRS R², representing the variance explained by the PRS after accounting for covariates, was observed when adjusting for sex only. The highest Null R^2^ was observed with age, sex, and ANC, indicating they explained the most variability independent of polygenic score. In contrast, the lowest Null R² was seen in the model including only sex, suggesting this alone has a limited contribution to the model variance and cannot be used alone to predict risk. The models that included age or sex, or both had statistically significant empirical *p*-values. Models with ANC alone or sex and ANC showed nominal significance, while the age, sex, and ANC as well as the age and ANC models were not significant. However, the magnitude of variance explained by genetic and covariate components varied depending on the covariate combination.

For Kim *et al*., 2024, the same seven covariate combinations were assessed. The highest PRS R^2^, showing the variance explained by the PRS model only, was observed when adjusting for sex (PRS R^2^ = 0.0552), while the lowest was seen when adjusting for age and ANC (PRS R^2^ = 0.0139). The Null R^2^ values are consistent with those observed in the EUR-based summary statistics, as expected, since they capture the variance explained by covariates alone and are independent of genetic influence. Finally, the variance explained by the PRS was dependent on the covariate structure, highlighting the impact of covariate selection on model performance.

We evaluated whether the inclusion of PRS improved the predictive performance for the models including only covariates (**Table 3**). For this, we looked at both base dataset summary statistics as well as the training and validation cohorts. The addition of the PRS consistently increased the AUC for all covariate combinations. The improvement was more pronounced in models with fewer covariates showing statistical significance (DeLong *p*-value <0.05). For models including the full set of covariates (age, sex, and ANC), the PRS still increased the AUC, though the change was smaller and, in some cases, not statistically significant (DeLong *p*-value >0.05). These results indicate that the PRS provides a meaningful addition to the predictive power beyond the covariates included.

**Table 3:** Effect of adding the polygenic risk score on predictive performance of covariate models.

Covariates	AUC (Covariates only)	AUC (Full model: Covariates + PRS)	DeLong p-value
**Training: Nalls *et al*., 2019**			
AGE	0.7645	0.7747	0.0147
SEX	0.5403	0.6060	1.09E-07
AGE + SEX	0.7700	0.7788	0.0196
AGE + ANC	0.8145	0.8161	0.4803
SEX + ANC	0.6364	0.6542	3.45E-03
ANC	0.6218	0.6437	0.0141
AGE + SEX + ANC	0.8159	0.8180	0.2848
**Validation: Nalls *et al*., 2019**			
AGE	0.7511	0.7694	0.0332
SEX	0.5863	0.6298	0.0136
AGE + SEX	0.7688	0.7832	0.0787
AGE + ANC	0.7909	0.8005	0.1869
SEX + ANC	0.6399	0.6680	0.0696
ANC	0.5942	0.6426	0.0371
AGE + SEX + ANC	0.7996	0.8085	0.2051
**Testing: Kim *et al*., 2024**			
AGE	0.7645	0.7789	3.10E-03
SEX	0.5403	0.6265	6.76E-06
AGE + SEX	0.7700	0.7840	3.55E-03
AGE + ANC	0.8145	0.8183	0.2560
SEX + ANC	0.6364	0.6731	4.27E-03
ANC	0.6218	0.6631	3.30E-03
AGE + SEX + ANC	0.8159	0.8213	0.1305
**Validation: Kim *et al*., 2024**			
AGE	0.7511	0.7847	0.0036
SEX	0.5863	0.6493	0.0028
AGE + SEX	0.7688	0.7956	0.0110
AGE + ANC	0.7909	0.8126	0.0435
SEX + ANC	0.6399	0.6830	0.0175
ANC	0.5942	0.6552	0.0166
AGE + SEX + ANC	0.7996	0.8199	0.0424

Legend: Comparison of model discrimination with and without the polygenic risk score (PRS). For each set of covariates, we report the area under the ROC curve (AUC) for the covariates-only model and the full model including the PRS, along with the p-value from the DeLong test. Significant p-values indicate that inclusion of the PRS significantly improves model discrimination beyond the covariates alone. ANC, Ancestral components

### Polygenic risk score analysis: age at onset

The PRS explained a small proportion of variance in PD AAO across all datasets. Specifically, for the Nalls *et al*. (2019) analysis, the PRS R² was 0.0019 in the training subset and 0.0054 in the validation subset. For the Kim *et al*. (2024) analysis, the PRS R² was 0.0005 in the training subset and 0.0243 in the validation subset. Overall, these results suggest that the selected PRS have limited explanatory power for AAO in this cohort.

### Internal polygenic risk score analysis using South African summary statistics

We constructed an internal PRS for PD using 351 variants that reached a suggestive significance threshold in our South African GWAS. After LD clumping, 141 independent variants remained and were included in the PRS. In the training cohort, the PRS explained 33.9% of the phenotypic variance (R² = 0.3386) and achieved an AUC of 0.7736. When applied to the validation cohort, the PRS explained 36.0% of the variance (R² = 0.3598) with an AUC of 0.7667, indicating consistent discriminative performance across cohorts.

## Discussion

To our knowledge, this is the first study to evaluate PRS for PD prediction in a South African cohort. We used a well-established PRS software, PRSice-2, and leveraged summary statistics from both EUR-ancestry and multi-ancestry GWAS for PD. Despite the summary statistics not fully matching the genetic background of our cohort, which is five-way admixed [16], our findings demonstrate that polygenic models can still capture a modest but significant proportion of the variance in PD susceptibility, highlighting the utility of PRS in diverse populations.

Using PRSice-2, we identified the best parameter sets for both the Nalls *et al*. (2019) and Kim *et al*. (2024) summary statistics. In the training dataset, we found that clumping parameters (e.g., r² and window size) and *p*-value thresholds had notable effects on predictive power. The optimal PRS derived from the Nalls *et al*. (2019) summary statistics achieved an AUC of 0.6077. Similarly, using the Kim *et al*. (2024) summary statistics with more diverse populations, the best-performing PRS model had an AUC of 0.6183. Although the PRS derived from Kim *et al*. (2024) explained more variance in disease risk, its slightly higher AUC compared to Nalls *et al*. (2019) highlights how R² and AUC capture different aspects of predictive performance, the underlying genetic predisposition versus classification accuracy, respectively. This may suggest that multi-ancestry meta-analysis PRS better models genetic predisposition for distinguishing cases from controls in the study population. However, the observed AUC is similar to those previously reported for PD PRS which range from 0.620 to 0.760 [15].

Notably, the models that achieved the highest predictive accuracy tended to favor moderate clumping thresholds and relatively lenient SNP inclusion thresholds, likely balancing the trade-off between including informative variants and controlling for LD-induced noise. Moreover, the validation of model performance in an independent subset of the cohort strengthens the robustness of these findings and supports the potential clinical utility of PRS in diverse populations.

Overall, our results highlight that while sex contributes modestly to the explained variance, accounting for approximately 3–5% across models, it cannot be used in isolation to predict disease status [15], as evidenced by the low full model R^2^ values when sex was the only covariate. In contrast, age appears to provide a more meaningful contribution, particularly when combined with other covariates such as ancestry. Importantly, the PRS R^2^ values were consistently low across all models and datasets, suggesting that genetic risk alone, as captured by current PRS methods, is insufficient for reliable disease prediction [18]. However, the DeLong test demonstrated that adding PRS to models with sex or ancestry alone can significantly improve AUC, whereas the incremental gain was not significant when age and ancestry covariates were already included. This underscores that the predictive contribution of PRS is dependent on demographic and ancestral factors. These findings underscore the importance of incorporating non-genetic covariates to enhance predictive performance, as previously illustrated in a PD context [19]. Ultimately, the limited variance explained by PRS alone constrains its current clinical utility for PD and emphasizes the need for integrative models that include both genetic and phenotypic information [8].

The additional sensitivity and specificity analyses support the overall performance of our PRS models, showing results that are consistent with previous work on multi-ancestry populations [20]. Our AUC values ranged from 0.5847 to 0.6183 across base datasets, with corresponding balanced accuracy values between 0.5343 and 0.5856. These values are comparable to those previously reported, whose AUC ranged from 0.505 to 0.651 across four ancestries [20]. Our model performs similarly to theirs, particularly in populations with higher predictive power, such as the EUR populations. Our sensitivity and specificity values also reflect expected trade-offs: for example, when sensitivity increased, specificity tended to decrease, consistent with typical classification dynamics. Together, these findings reinforce the idea that PRS models retain moderate predictive power in diverse populations, but that further optimization may be needed to improve performance, especially in underrepresented groups. This aligns with previous reports showing PRS transferability from EUR to AFR populations is substantially reduced, with performance estimated at 20–40% of that observed in EUR populations [21].

We generated an internal PRS using summary statistics from our South African GWAS. Of 351 suggestively associated variants, 141 remained after clumping. The PRS explained 33.9% of the variance in the training cohort (AUC = 0.774) and 36.0% in the validation cohort (AUC = 0.767). As this PRS was derived and tested in the same cohort, these estimates likely reflect overfitting and should be interpreted as cohort-specific rather than generalizable predictive performance.

Despite the moderate predictive performance observed, our study underscores both the promise and limitations of current PRS models in underrepresented populations. A key limitation of this study is the small sample size and the lower mean age of our control group relative to the case group. A further limitation for the study is the subsequent lack of an appropriate ancestry-matched validation cohort. Moreover, the AUC values achieved reflect modest discriminative power. This finding suggests that while PRS can contribute to risk stratification, they are not yet sufficient for clinical decision-making on their own. This is further illustrated by the AAO PRS, which explains only a modest proportion of variation in AAO, highlighting its limited predictive power for this phenotype. Future studies incorporating ancestry-specific GWAS, functional annotations, and integrative risk models may further improve PRS accuracy in AFR and admixed populations. As LD resources tailored to AFR and admixed genomes continue to improve, evaluating LD-aware methods such as PRS-CS or PRS-CSx will be critical to determine whether these approaches can further enhance PRS performance in this setting [22,23].

A key strength of the study is its novelty, representing the first evaluation of PRS for PD in a South African study collection, thereby addressing a critical gap in genetic risk research. By including both EUR-based and multi-ancestry summary statistics, we were able to compare the PRS transferability across ancestries and assess how base dataset ancestral composition influences predictive performance. Furthermore, the systematic evaluation of clumping thresholds, LD parameter, and *p*-value thresholds to identify the optimal input parameter combinations further strengthens the methodological approach of this analysis. Finally, the application of PRS across various diseases, including diabetes and cancer [24,25], has proven valuable for stratifying individuals at highest risk, rather than serving as a direct predictor of disease development [26]. In this context, our study contributes by refining PD risk prediction for a smaller subset of individuals most at risk for developing PD.

In conclusion, our results highlight the importance of including diverse ancestral cohorts and relevant covariates when constructing and evaluating PRS models. By systematically assessing the variance observed across different covariate combinations, we highlight the contributions of demographic and genetic factors to disease risk prediction. Future efforts should continue to refine ancestry specific risk-models to ensure equitable translation of PRS from research into clinical applications for early screening, disease risk prediction, and precision medicine [27].

## Materials and methods

### Ethics statement

Ethical approval was obtained from the Health Research Ethics Committee 1 (HREC 1), Stellenbosch University, South Africa. The HREC 1 reference number is: S23/10/251 (PhD) Sub Study 2002C/059. All participants provided a written statement of formal consent.

### Participant demographics

Study participants were recruited from 2002 to 2020 as part of the South African PD Study Collection (**S2 Table**) [28]. Individuals living with PD (n = 691) were diagnosed in accordance with the Queen’s Square Brain Bank Criteria [29]. In total, 826 controls were recruited as part of the study collection [28].

### Data preprocessing

Genotyping was completed through the Global Parkinson’s Genetics Program [30] using the NeuroBooster Array (v1.0, Illumina, San Diego, CA) [31]. Quality control (QC) was performed using PLINK v1.9 and v2.0 [32,33], as previously described [16]. Imputation was performed using the TOPMed Imputation Server [34]. The related individuals (n = 63) were identified using NAToRA [35] and a kinship coefficient of 0.0884 (second degree relation [36]) and excluded from the analysis. After QC, 661 PD cases and 737 controls remained (**S2 Table**). The South African population is five-way admixed [37], therefore, a reference panel was created using samples from the 1000 Genomes Project Phase III, including individuals of African (AFR), EUR, and South Asian (SAS) ancestries [38,39]. Additional individuals of Malay (MAL) ancestry and an indigenous hunter-gatherer Khoe-San (NAMA) population were included in the reference panel [40], as previously described [16]. The reference files were phased using the TOPMed Imputation Server [34]. For the South African dataset, local ancestry inference was performed using G-Nomix [41], as previously described [16] (**S6 Fig**).

### Polygenic risk score calculation

**Fig 2:**
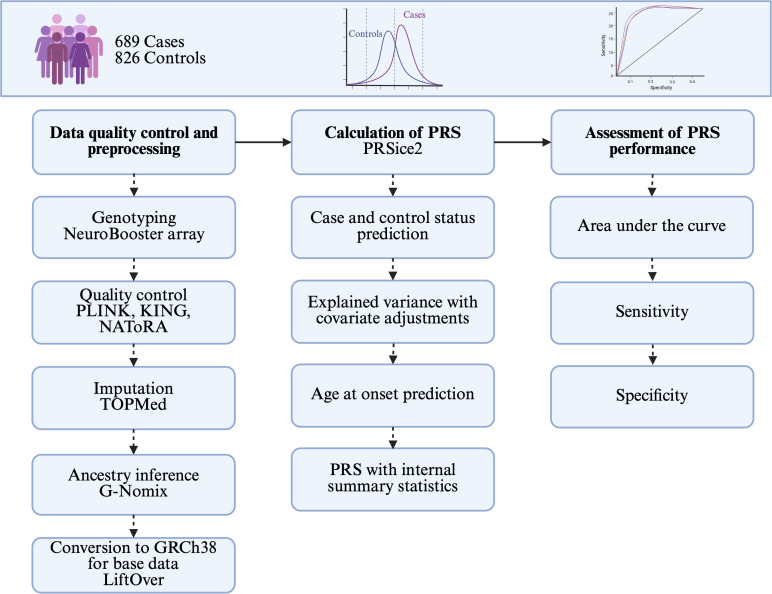
An overview of the methods followed in the present study. LiftOver was specific to the summary statistics used as the base datasets. PRS, polygenic risk score. Image created with BioRender.com/. https://BioRender.com/19qh9dv.

A PRS analysis includes the following steps (**Fig 2**): [1] data QC and preparation, [2] PRS calculation, and [3] PRS performance assessment [9]. The analysis utilizes two independent datasets: discovery and target datasets [9]. The discovery dataset consists of GWAS summary statistics, including effect sizes for each variant. The target dataset contains individual-level genotype data, from which SNP dosages are derived for variants included in the PRS calculation. In general, PRS is computed for each individual as the sum of the dosages of risk alleles at selected SNPs, weighted by their corresponding effect sizes from the discovery dataset [42]. The PRS analysis was run using PRSice-2 v2.3.3 [43], which applies clumping and thresholding based on LD and *p*-values. The predictive PRS model is evaluated using Nagelkerke’s pseudo-R^2^ (R^2^) [9].

### Polygenic risk score: file preparation

The covariate file was created using the local ancestry analysis output containing the ancestry inferences for the parental populations for each individual and both haplotypes across inferred local ancestry windows. The ancestry proportions were calculated by extracting the ancestry window information, specifically calculating the total genomic span of each parental ancestry, and normalizing the values to determine relative ancestry contribution per individual. Additional covariates included age and sex. Full summary statistics were obtained from the NHGRI-EBI GWAS catalog [44] on 05/09/2024 for studies GCST009325 [7] and GCST90275127 [17]. These two base datasets were used to assess and compare the predictive power of the EUR dataset relative to a multi-ancestry dataset which is better matched to the admixture of the South African cohort. This approach was used to evaluate whether ancestry-matched summary statistics enhanced predictive performance in an admixed population, as traditional EUR-derived GWAS does not fully capture the genetic architecture in diverse populations, like the South African population. The summary statistics were converted to GRCh38 using LiftOver v1.0 [45]. The South African PD data, serving as the target dataset, was randomly split into two cohorts: 70% of the samples were in the training dataset (n = 979 individuals; n = 445 cases; n = 534 controls), and 30% in the validation dataset (n = 419 individuals; n = 216 cases; n = 203 controls). To assess the robustness of the data split, we ran the PRS analysis across 20 random splits and compared the distribution of the AUC values with the original split (**S3 Table**).

### Polygenic risk score analysis: case status

For this analysis, clumping was conducted using window sizes of 100kb, 250kb, and 500kb; LD thresholds (r^2^) of 0.1, 0.2, 0.5, and 0.8; as well as the *p*-value thresholds of 1 × 10^-3^, 1 × 10^-5^, 1 × 10^-6^, and 5 × 10^-8^. The 1000 Genomes EUR reference panel was used for LD calculations in both base datasets, as the Nalls et al. (2019) study is EUR-based and approximately 62% of participants in the Kim et al. (2024) study were of EUR ancestry [7,17,46]. All analyses were performed using logistic regression, adjusting for sex, age, and ancestral components (AFR, EUR, NAMA, SAS). To prevent perfect multicollinearity, we excluded the MAL ancestry from the covariates, as it represented the smallest ancestral contribution. To obtain robust significance estimates, empirical *p*-values were calculated using 10,000 phenotype permutations. The initial search for optimal PRS parameters was conducted on 70% of the dataset (training cohort), and the best-performing parameters and variants included in the model were then applied to fit a new model on the remaining 30% (validation cohort). The AUC was used to evaluate the performance of each PRS model using the pROC package [47] in R v4.2.0, providing a quantitative metric for comparing models [48]. Additionally, predicted probabilities from a logistic regression model were converted to binary disease status using a fixed threshold of 0.5, and model performance was evaluated using accuracy, balanced accuracy, sensitivity, and specificity calculated at this threshold. Additionally, the positive predictive value and negative predictive values were calculated at multiple top-percentile thresholds (5%, 10%, and 20%) using the global PD prevalence of 1.386 × 10^-4^ [49].

### Assessment covariate variance

We evaluated the contribution of different covariate combinations to the variance explained. Using PRSice-2 outputs, we examined the variance models across seven covariate inclusion scenarios, each incorporating a distinct combination of the following variables: age, sex, and ANC. This analysis allowed us to quantify the incremental variance explained by each covariate and their combinations, providing insight into their effects on risk prediction.

### Polygenic risk score analysis: age at onset

PRS for PD age at onset (AAO) were generated using PRSice-2. The analysis followed the same 70/30 training-validation split and identical PRS parameters described above, with the only modification being that AAO was modelled as a continuous phenotype rather than a binary outcome (as seen in the PRS for case status). The aforementioned covariates were used and LD was calculated with the EUR reference panel. This analysis was performed using both base datasets as well as the training and validation cohorts.

### Internal polygenic risk score analysis using South African summary statistics

We constructed a PD PRS using summary statistics from our internal GWAS [16]. Variants were selected based on a suggestive significance threshold (p < 5 × 10 ⁻ ⁶) to capture loci with potential contribution to PD risk within this population, and to see how predictive performance varies in comparison to the external summary statistics. The analysis was performed in the same 70.30 split using the optimal thresholds determined above. Predictive performance of the PRS was assessed by calculating the AUC.

## Supporting information

S1 Fig The following plots are using Nalls *et al* 2019 as the base dataset. (A) PRS R² across clumping thresholds. The variance explained (R²) by the polygenic risk score at different GWAS p-value thresholds, stratified by linkage disequilibrium clumping parameters (r² and kb). Each panel corresponds to a clumping window size (kb), with points and lines indicating R² across p-value thresholds. (B) PRS significance versus predictive performance. Relationship between PRS model significance (PRS association p-value; log-scaled) and variance explained (R²). Colors indicate clumping window sizes (kb) and shapes indicate LD thresholds (r²), highlighting the trade-off between model fit and predictive power.
(TIFF)

S2 Fig The following plots are using Kim *et al* 2024 as the base dataset. (A) PRS R² across clumping thresholds. The variance explained (R²) by the polygenic risk score at different GWAS p-value thresholds, stratified by linkage disequilibrium clumping parameters (r² and kb). Each panel corresponds to a clumping window size (kb), with points and lines indicating R² across p-value thresholds. (B) PRS significance versus predictive performance. Relationship between PRS model significance (PRS association p-value; log-scaled) and variance explained (R²). Colors indicate clumping window sizes (kb) and shapes indicate LD thresholds (r²), highlighting the trade-off between model fit and predictive power.
(TIFF)

S3 Fig Density plots showing the polygenic risk score distribution for cases versus controls. (A) Density plot with Nalls *et al* 2019 as the base dataset and (B) the density plot with Kim *et al* 2024.
(TIFF)

S4 Fig Bars plot showing the proportion of phenotypic variance (R²) explained by the polygenic risk score (PRS) at varying SNP inclusion p-value thresholds. Each bar represents a PRS model fit calculated at a specific threshold. The optimal threshold (defined as the point with the highest R²) is highlighted, indicating the most predictive model. (A) Results shown are based on the training dataset using summary statistics from Nalls *et al*., 2019 for the strongest association. (B) Results shown are based on the training dataset using summary statistics from Nalls *et al*., 2019 for the highest predictive performance. (C) Results shown are based on the validation dataset using summary statistics from Nalls *et al*., 2019 based on the highest predictive performance thresholds from the training dataset.
(TIFF)

S5 Fig Bars plot showing the proportion of phenotypic variance (R²) explained by the polygenic risk score (PRS) at varying SNP inclusion p-value thresholds. Each bar represents a PRS model fit calculated at a specific threshold. The optimal threshold (defined as the point with the highest R²) is highlighted, indicating the most predictive model. (A) Results shown are based on the training dataset using summary statistics from Kim *et al*., 2019 for the strongest association. (B) Results shown are based on the training dataset using summary statistics from Kim *et al*., 2024 for the highest predictive performance. (C) Results shown are based on the validation dataset using summary statistics from Kim *et al*., 2024 based on the highest predictive performance thresholds from the validation dataset.
(TIFF)

S6 Fig Karyograms showing the inferred local ancestry of three individuals from the South African Parkinson’s Disease Study Collection. This highlights the complex admixture of the cohort. AFR, African; EAS, East Asian; EUR, European; MAL, Malaysian; NAMA, Nama; POP, Population; SAS, South Asian.
(TIFF)

S1 Table Positive predictive value and negative predictive value of polygenic risk scores for Parkinson’s disease at multiple top-percentile thresholds. (XLSX)

S2 Table Study participants included in the polygenic risk score analysis. (XLSX)

S3 Table Stability of PRS performance estimates across repeated for 20 random splits. (XLSX)

S4 Table Global Parkinson’s Genetics Program banner author list. (XLSX)
