# Supplementary figures and images for: Polygenic risk scores and Parkinson’s disease in South Africa advancing ancestry informed disease prediction
Source: PLoS Genet. 2026 Mar 9;22(3):e1012064. doi: 10.1371/journal.pgen.1012064 (PMC12987585; doi:10.1371/journal.pgen.1012064)

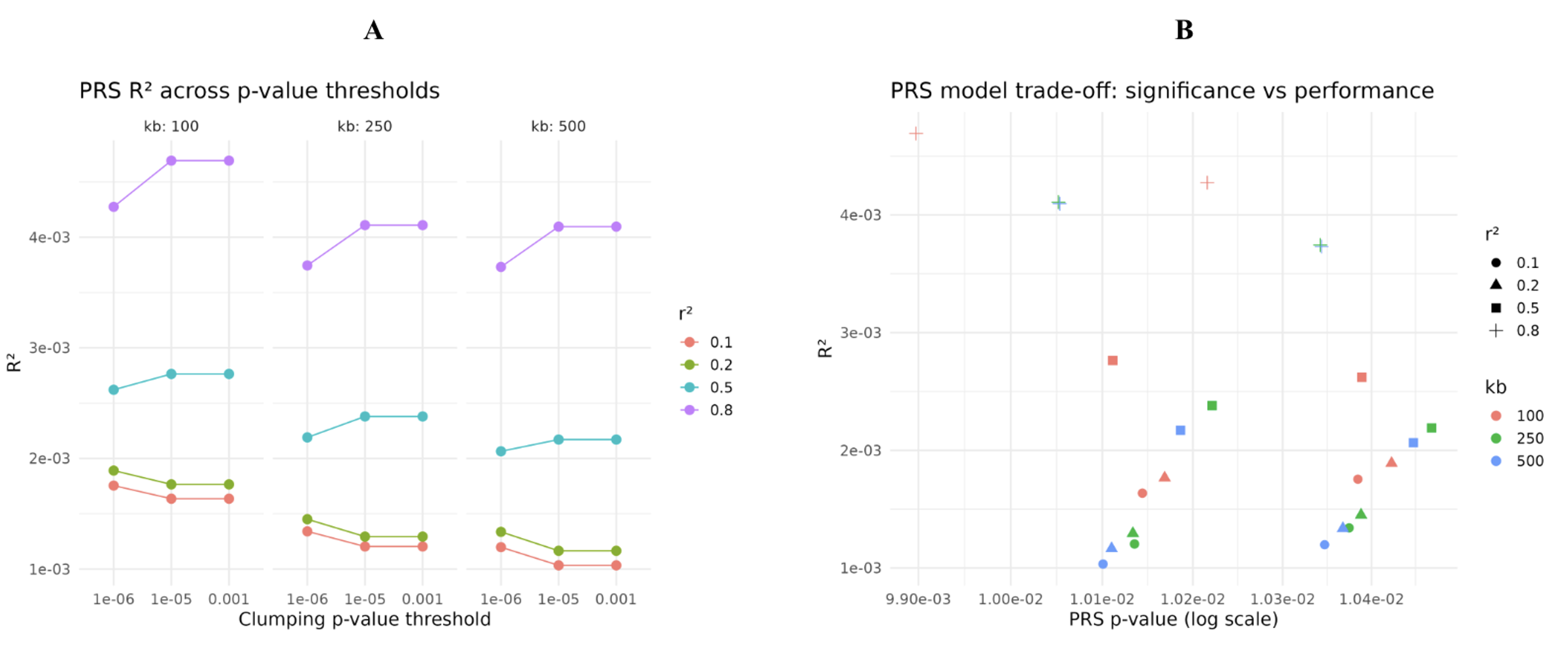

Supplement: S1 Fig — (A) PRS R² across clumping thresholds. The variance explained (R²) by the polygenic risk score at different GWAS p-value thresholds, stratified by linkage disequilibrium clumping parameters (r² and kb). Each panel corresponds to a clumping window size (kb), with points and lines indicating R² across p-value thresholds. (B) PRS significance versus predictive performance. Relationship between PRS model significance (PRS association p-value; log-scaled) and variance explained (R²). Colors indicate clumping window sizes (kb) and shapes indicate LD thresholds (r²), highlighting the trade-off between model fit and predictive power. (TIFF) [file pgen.1012064.s001.tiff]

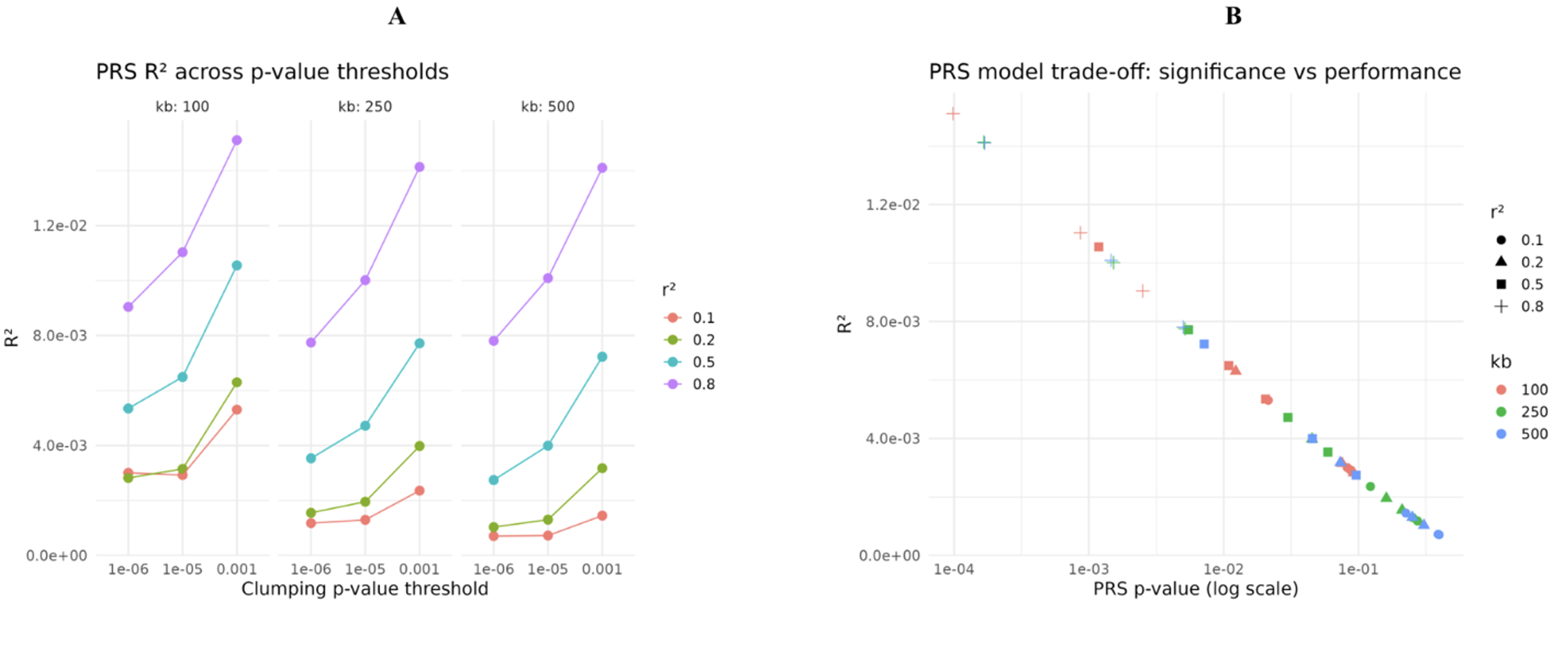

Supplement: S2 Fig — (A) PRS R² across clumping thresholds. The variance explained (R²) by the polygenic risk score at different GWAS p-value thresholds, stratified by linkage disequilibrium clumping parameters (r² and kb). Each panel corresponds to a clumping window size (kb), with points and lines indicating R² across p-value thresholds. (B) PRS significance versus predictive performance. Relationship between PRS model significance (PRS association p-value; log-scaled) and variance explained (R²). Colors indicate clumping window sizes (kb) and shapes indicate LD thresholds (r²), highlighting the trade-off between model fit and predictive power. (TIFF) [file pgen.1012064.s002.tiff]

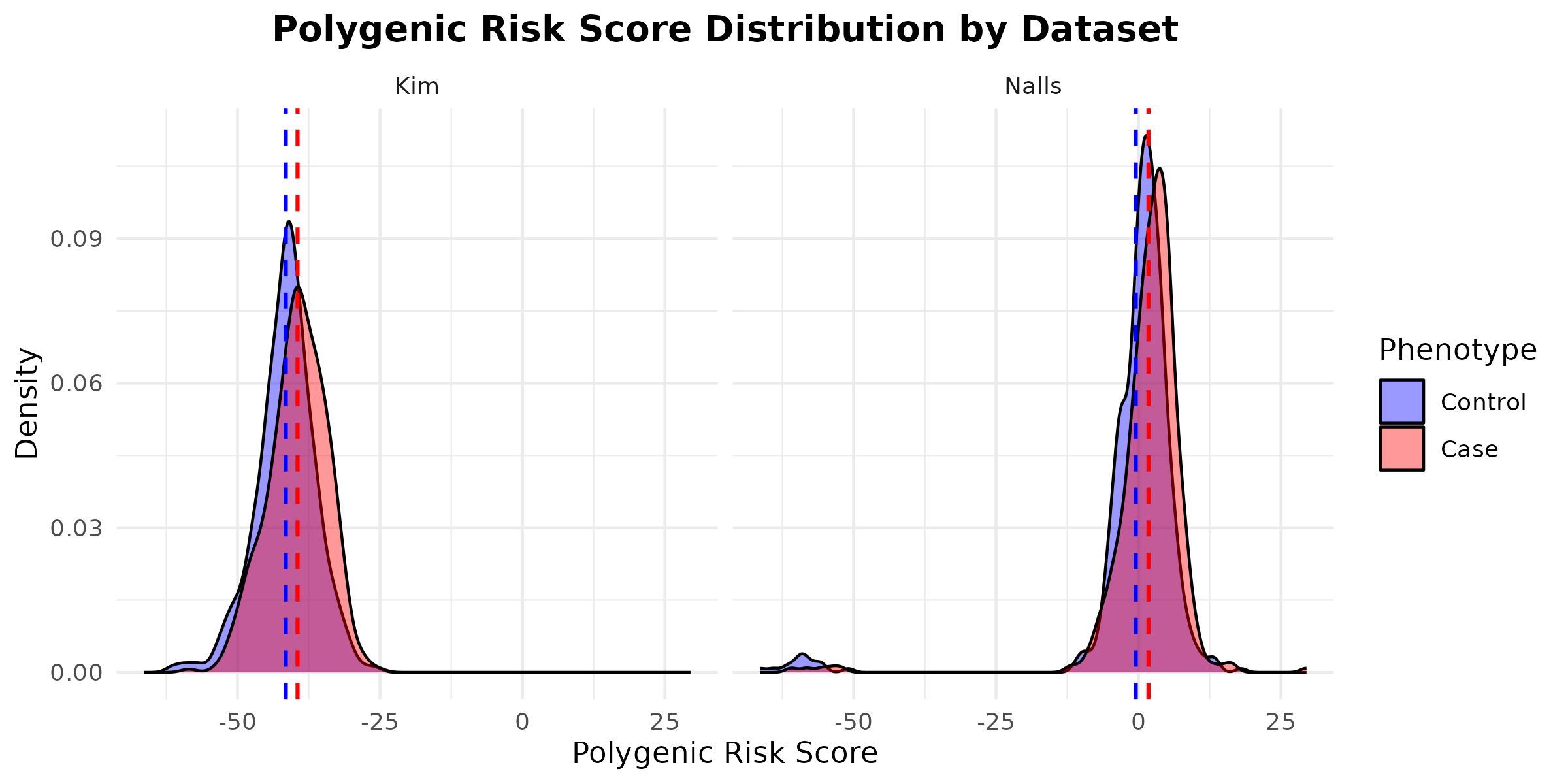

Supplement: S3 Fig — (A) Density plot with Nalls et al 2019 as the base dataset and (B) the density plot with Kim et al 2024. (TIFF) [file pgen.1012064.s003.tiff]

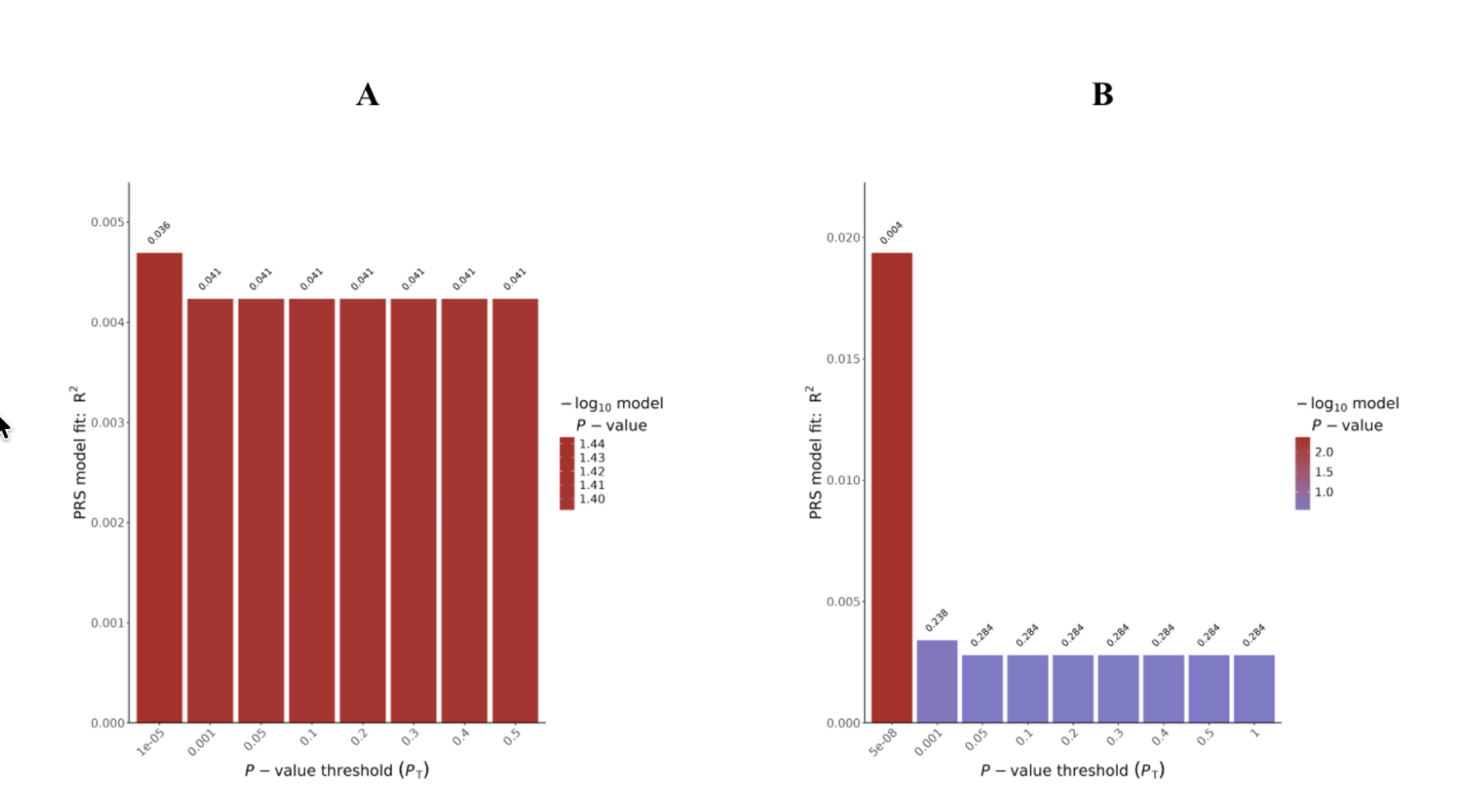

Supplement: S4 Fig — Each bar represents a PRS model fit calculated at a specific threshold. The optimal threshold (defined as the point with the highest R²) is highlighted, indicating the most predictive model. (A) Results shown are based on the training dataset using summary statistics from Nalls et al., 2019 for the strongest association. (B) Results shown are based on the training dataset using summary statistics from Nalls et al., 2019 for the highest predictive performance. (C) Results shown are based on the validation dataset using summary statistics from Nalls et al., 2019 based on the highest predictive performance thresholds from the training dataset. (TIFF) [file pgen.1012064.s004.tiff]

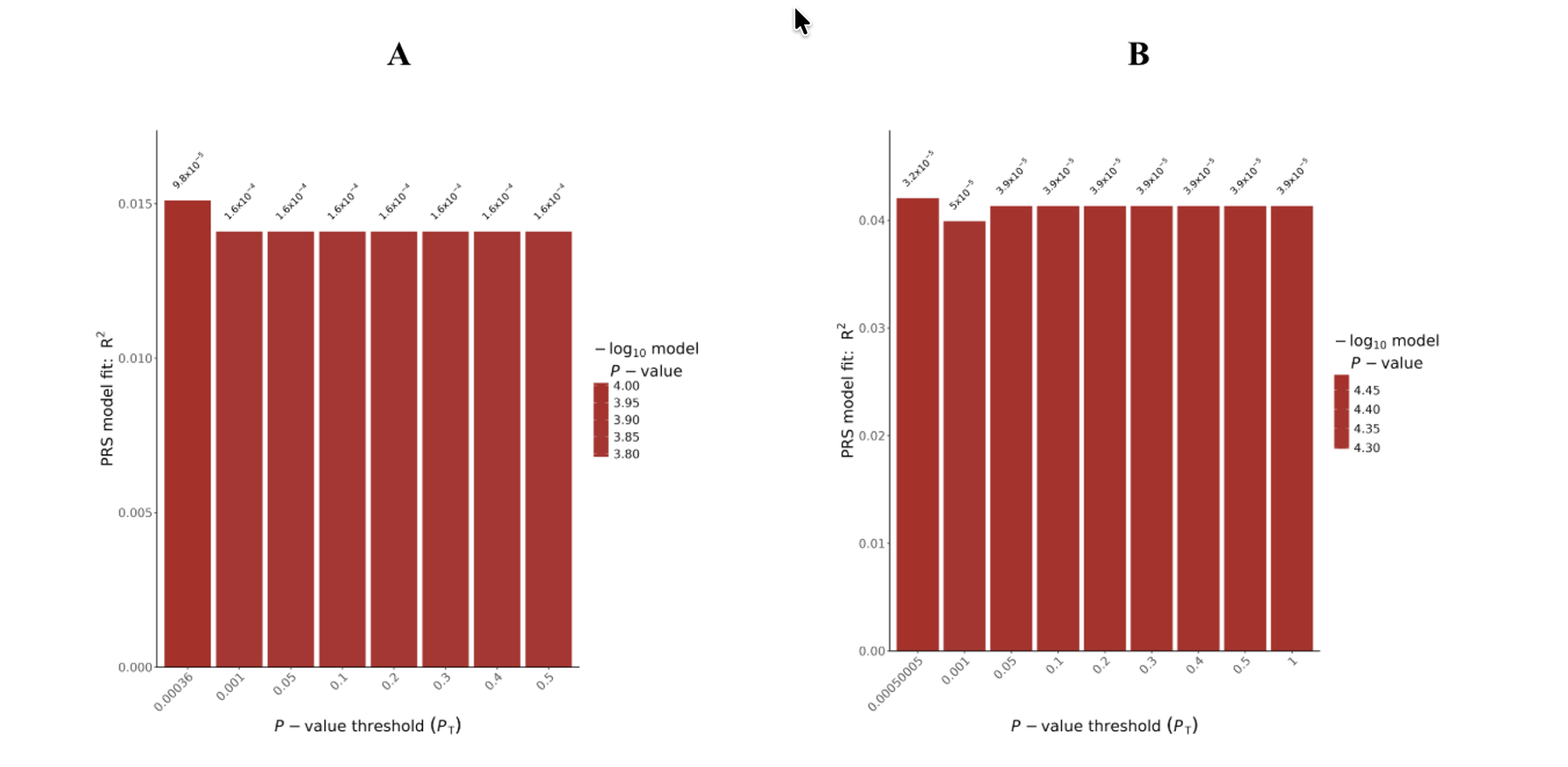

Supplement: S5 Fig — Each bar represents a PRS model fit calculated at a specific threshold. The optimal threshold (defined as the point with the highest R²) is highlighted, indicating the most predictive model. (A) Results shown are based on the training dataset using summary statistics from Kim et al., 2019 for the strongest association. (B) Results shown are based on the training dataset using summary statistics from Kim et al., 2024 for the highest predictive performance. (C) Results shown are based on the validation dataset using summary statistics from Kim et al., 2024 based on the highest predictive performance thresholds from the validation dataset. (TIFF) [file pgen.1012064.s005.tiff]

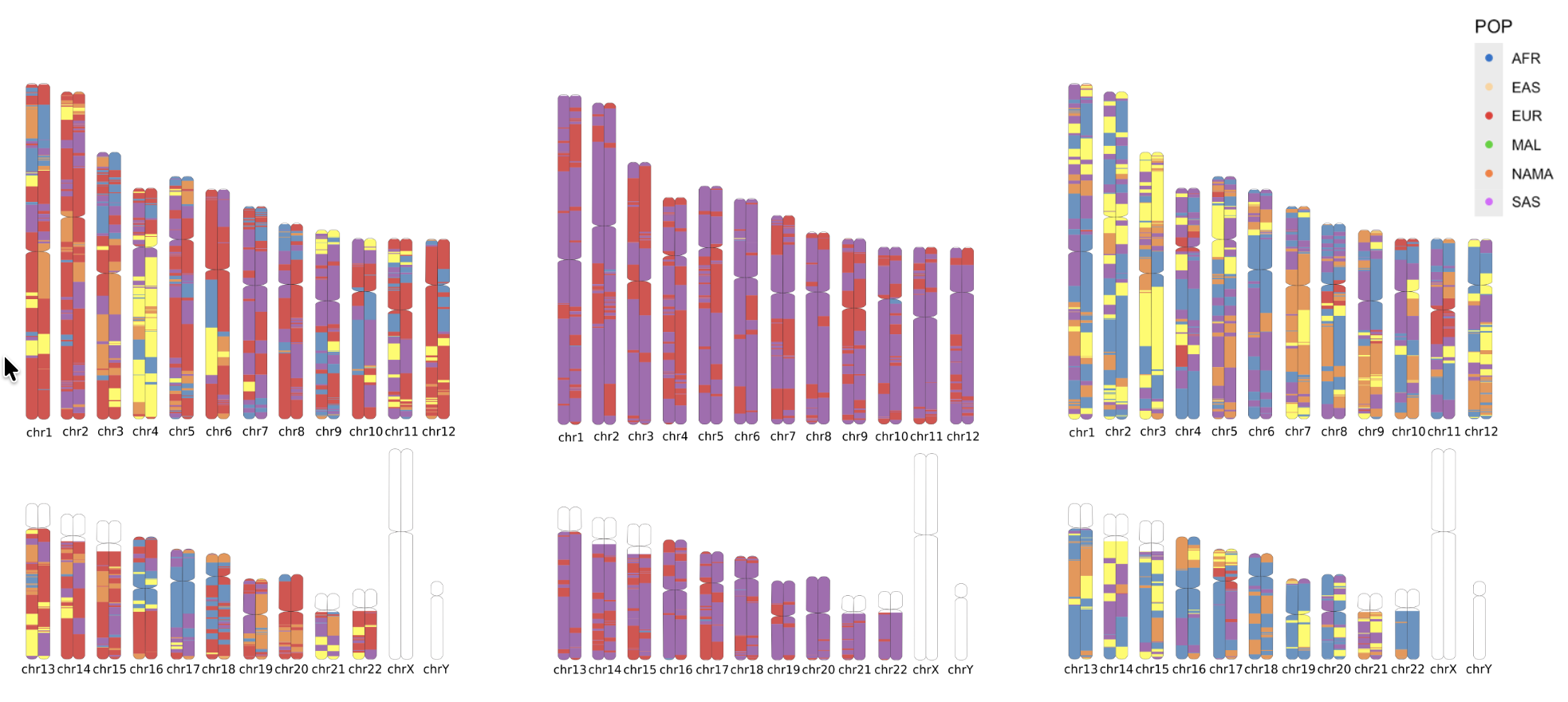

Supplement: S6 Fig — This highlights the complex admixture of the cohort. AFR, African; EAS, East Asian; EUR, European; MAL, Malaysian; NAMA, Nama; POP, Population; SAS, South Asian. (TIFF) [file pgen.1012064.s006.tiff]
